# Impact of the Negative Pressure Wound Therapy System (PICO 7 Smith and Nephew) on Surgical Site Infections in High-Risk Patients Undergoing Elective Colorectal Resections and Emergency Laparotomy

**DOI:** 10.7759/cureus.77103

**Published:** 2025-01-07

**Authors:** Abhijeet Beniwal, Isha Karwasra

**Affiliations:** 1 Colorectal Surgery, St. Mark's Hospital at Central Middlesex Hospital, London, GBR; 2 Surgery, University Hospital Southampton NHS Foundation Trust, Southampton, GBR

**Keywords:** colorectal surgery, national emergency laparotomy audit (nela), negative pressure woundtherapy, surgical site infection (ssi), emergency laparotomy

## Abstract

Background: Surgical site infections (SSIs) are a significant concern in colorectal surgery, impacting patient outcomes and increasing treatment costs. The study investigates the effect of PICO 7 (Smith & Nephew) dressings, a negative pressure wound therapy (NPWT) system, on the incidence of SSIs in high-risk patients undergoing elective colorectal resections and emergency laparotomies.

Methods: This retrospective cohort study was conducted at the Colorectal Surgery Unit at Russell’s Hall Hospital, Dudley, United Kingdom. The cohort included patients who underwent open colorectal resections and emergency laparotomies with PICO 7 dressing following laparotomy wound closure. The primary endpoint was the incidence of SSI within 30 days of surgery. Secondary endpoints included length of hospital stay, frequency of readmissions, episodes of return to theatre, and interventions such as radiological or open drainage of the abscess. Data were compared with existing literature, given the plan was originally to compare outcomes with a cohort managed with conventional dressings.

Results: A total of 27 cases were reviewed where PICO 7 was applied. Of these, 21 were emergency laparotomies, 11 patients had a stoma created, and 16 had abdominal cavity contamination (faecal matter, pus, serous collection). The majority (14 patients) had a body mass index (BMI) >30, and 16 patients had an American Society of Anesthesiologists (ASA) grade 3. Out of these, eight (30%) developed SSIs, including overlaps of dehiscence and deep space infection. The average hospital stay was 17 days, with one patient readmitted with SSI and four requiring interventions such as return to theatre or radiological drainage.

Conclusions: The study found a higher incidence of SSIs (30%) compared to existing literature, despite using NPWT. Contributing factors included perforated viscus, high BMI, multiple comorbidities, and stoma creation. A larger study cohort with a control group is necessary for further evaluation.

## Introduction

Surgical site infections (SSIs) are a significant burden on healthcare systems, ranking as the third most common nosocomial infection after urinary tract infections and hospital-acquired pneumonia in the United Kingdom (UK). In the United States (US), SSIs account for approximately 36.4% of infections reported to the Centers for Disease Control and Prevention (CDC) National Healthcare Safety Network (NHSN). Colorectal surgeries are particularly prone to SSIs, representing the second-highest risk after liver transplant procedures, with abdominal surgeries being the most common overall cause of SSIs [1-3].

SSIs significantly impact patient outcomes, prolonging hospital stays, increasing morbidity, and negatively affecting psychological well-being. For cancer patients, SSIs can delay adjuvant therapies, including chemotherapy and radiotherapy, thereby compromising oncological outcomes [4]. Beyond clinical consequences, SSIs impose considerable financial burdens on healthcare systems. In the US, the management of SSIs incurs over $1.6 billion in additional costs, including $1 million attributed to extended hospital stays [5]. Similarly, the National Health Service (NHS) in the UK faces significant expenses, with average costs estimated at £10,523 per SSI, encompassing both hospital and community care expenses [6]. These infections also result in substantial mortality, with up to 5,000 annual deaths in the NHS attributable to SSIs [7].

The risk of developing SSIs is influenced by multiple factors, including patient characteristics (e.g., diabetes, obesity, smoking, malnutrition, and immunosuppression) and surgical parameters (e.g., prolonged operative times, contamination from bowel contents, and intraoperative hypotension). High-risk populations, such as those with a body mass index (BMI) >30, an American Society of Anesthesiologists (ASA) physical status score >3, or undergoing emergency surgeries, are particularly vulnerable [8]. The incidence of SSIs in colorectal surgeries remains high, ranging from 15% to 30%, even with adherence to prophylactic antibiotics and aseptic techniques [9,10].

Several interventions have been introduced to mitigate SSI risk, including perioperative care bundles, appropriate antibiotic prophylaxis, and intraoperative measures such as wound irrigation, glycemic control, and the use of wound protectors [8,11]. However, these strategies are not universally effective, especially in colorectal procedures where challenging patient profiles and contaminated surgical fields are common. Among emerging techniques, negative pressure wound therapy (NPWT) has gained attention for its potential to reduce SSI rates through mechanisms such as reducing lateral tension, enhancing tissue perfusion, and removing exudates and infectious materials [12].

The PICO 7 NPWT device is designed for use in low to moderate exudate wounds, promoting healing via absorbent dressing technology coupled with negative pressure. While NPWT has shown promise in various surgical fields, its application in colorectal surgeries remains underexplored within the NHS. This study aims to evaluate the efficacy of PICO 7 NPWT in reducing SSIs, hospital stays, and associated costs in patients undergoing colorectal resections and emergency laparotomies.

## Materials and methods

Study design

A retrospective cohort study was conducted in the Colorectal Surgery Unit at Russell’s Hall Hospital, Dudley, UK. This study included patients who underwent open abdominal surgery and had an NPWT device (PICO Smith and Nephew, Watford, England) applied at the time of laparotomy wound closure between January 2020 and June 2020. Due to logistical challenges imposed by the SARS-CoV-2 pandemic, including restricted access to patient records, a planned comparison with a matched cohort of patients receiving conventional dressings was not feasible. Instead, the results were compared with existing literature.

Study population

PICO 7 dressings had been recently introduced at the NHS trust and were selectively applied to a limited patient group. PICO dressing was applied to the wounds after primary closure of wounds. All patients undergoing laparotomy with PICO 7 dressings were included, resulting in a small sample size. Initially, the study intended to analyze elective colorectal resections only. However, due to the low number of open colorectal resections, emergency laparotomies were also included to enhance the sample size.

The inclusion criteria and exclusion criteria are included in Table 1.

**Table 1:** Inclusion and exclusion criteria

Inclusion Criteria	Exclusion Criteria
Patient Age ≥18 years	Purely laparoscopic cases
Open abdominal surgery or converted laparoscopic procedures in elective and emergency settings between January 2020 and June 2020.	Pregnant patients
High-risk patients with BMI >35, diabetes mellitus, bowel malignancy, and class II, III, and IV wounds.	Patients, on chronic immunosuppressive therapy.
Elective colorectal resections included colorectal malignancy, complicated diverticular disease with fistulas, and inflammatory bowel diseases such as Crohn’s disease or ulcerative colitis.	Patients with allergies to iodine or adhesive drapes, or with prosthetic mesh or subcutaneous drains.
Emergency laparotomies included cases of perforation peritonitis, bowel obstruction, and bowel ischemia.	Patients with existing surgical site infections at surgery
	Patients who underwent re-surgery from the original incision within 30 days for non-wound-related complications.
	Patients lacking a 30-day follow-up.

Data collection

Patient records, including hospital notes, operative reports, and anesthesia notes, were reviewed. Wound classifications were based on the surgeon’s assessment in cases of discrepancies. Information on surgical site complications, antibiotic use, and hospital readmissions was collected. SSIs were defined based on CDC criteria. Data collection also included wound-related complications, hospital stay duration, and follow-up records from outpatient clinics, colorectal nurses, and tissue viability nurses.

Study endpoints

Primary endpoint: Incidence of SSI within 30 days of surgery, defined using CDC criteria [13].

Secondary endpoints: Metrics reflecting the impact of SSIs, including outpatient wound review visits, hospital stay length, readmission rates, re-operations, interventions for abscesses, and associated healthcare costs.

Ethical considerations

The research proposal was submitted to the Trust's Research and Development department for data access. Strict adherence to data confidentiality and GDPR guidelines was maintained, with all patient identifiers anonymised and data analysed solely within the hospital setting.

Statistical analysis

Statistical analyses were performed using Statistical Product and Service Solutions (SPSS, version 23; IBM SPSS Statistics for Windows, Armonk, NY). Tests included the Student's t-test, chi-square test, and Fisher’s exact test, with a significance threshold set at p<0.05.

## Results

Patient characteristics

**Figure 1:**
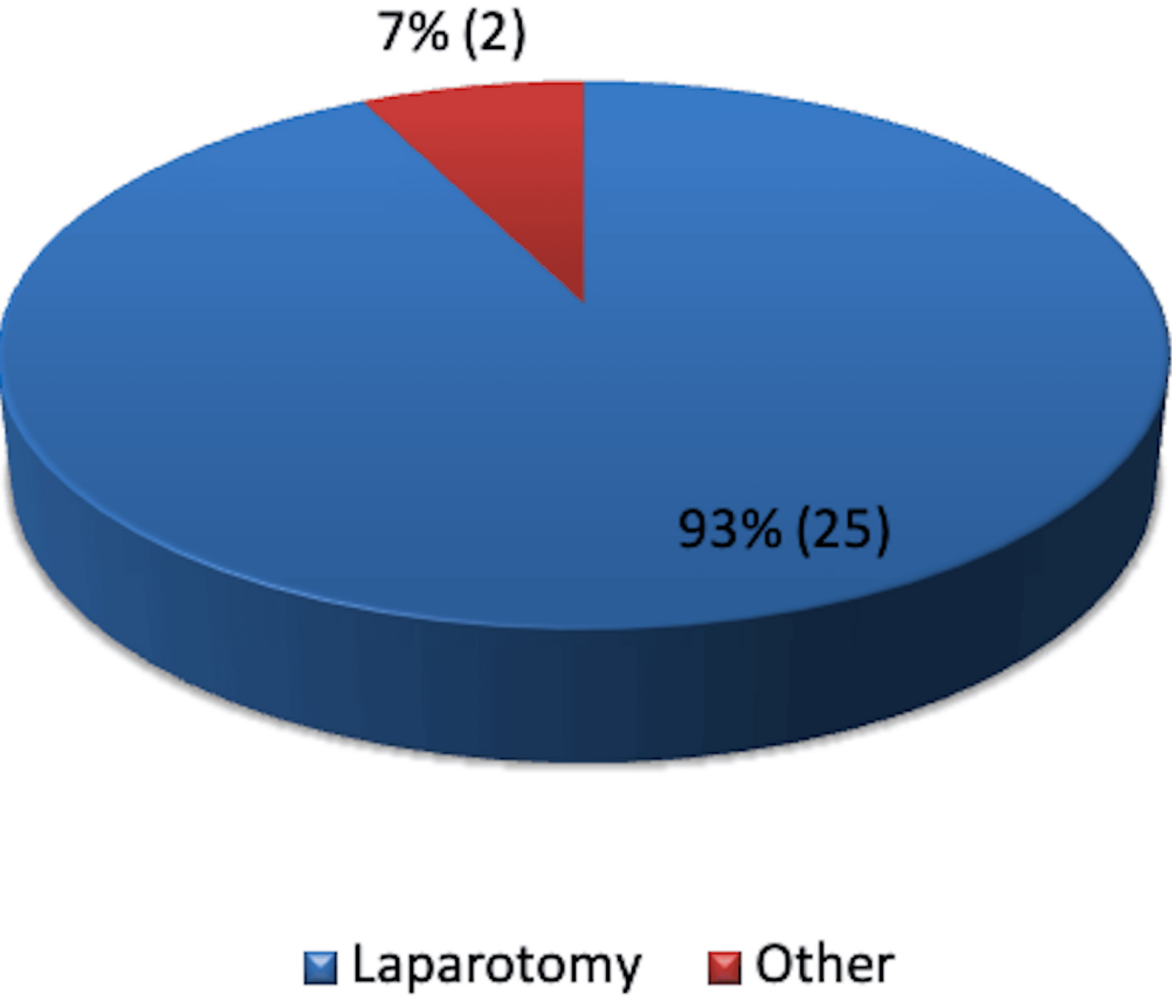
Type of surgery

The dataset comprised 26 patients who underwent surgical procedures between April 2019 and October 2019 with NPWT using PICO 7. Among these, 25 patients had a laparotomy, while one patient required two wound debridement procedures, accounting for the remaining two PICO cases (Figure 1).

**Figure 2:**
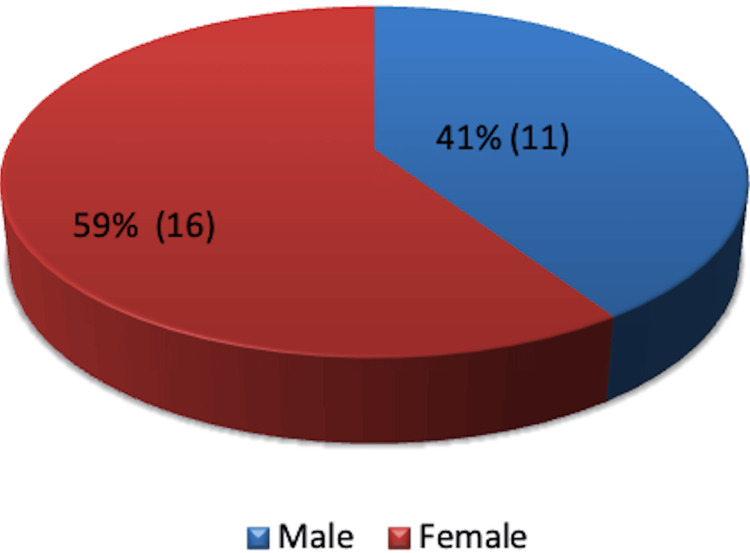
Gender distribution - male or female

The cohort included 11 males and 16 females, with a mean age of 63 years. The youngest patient was 36 years old, while the eldest was 89 years old (Figure 2).

**Figure 3:**
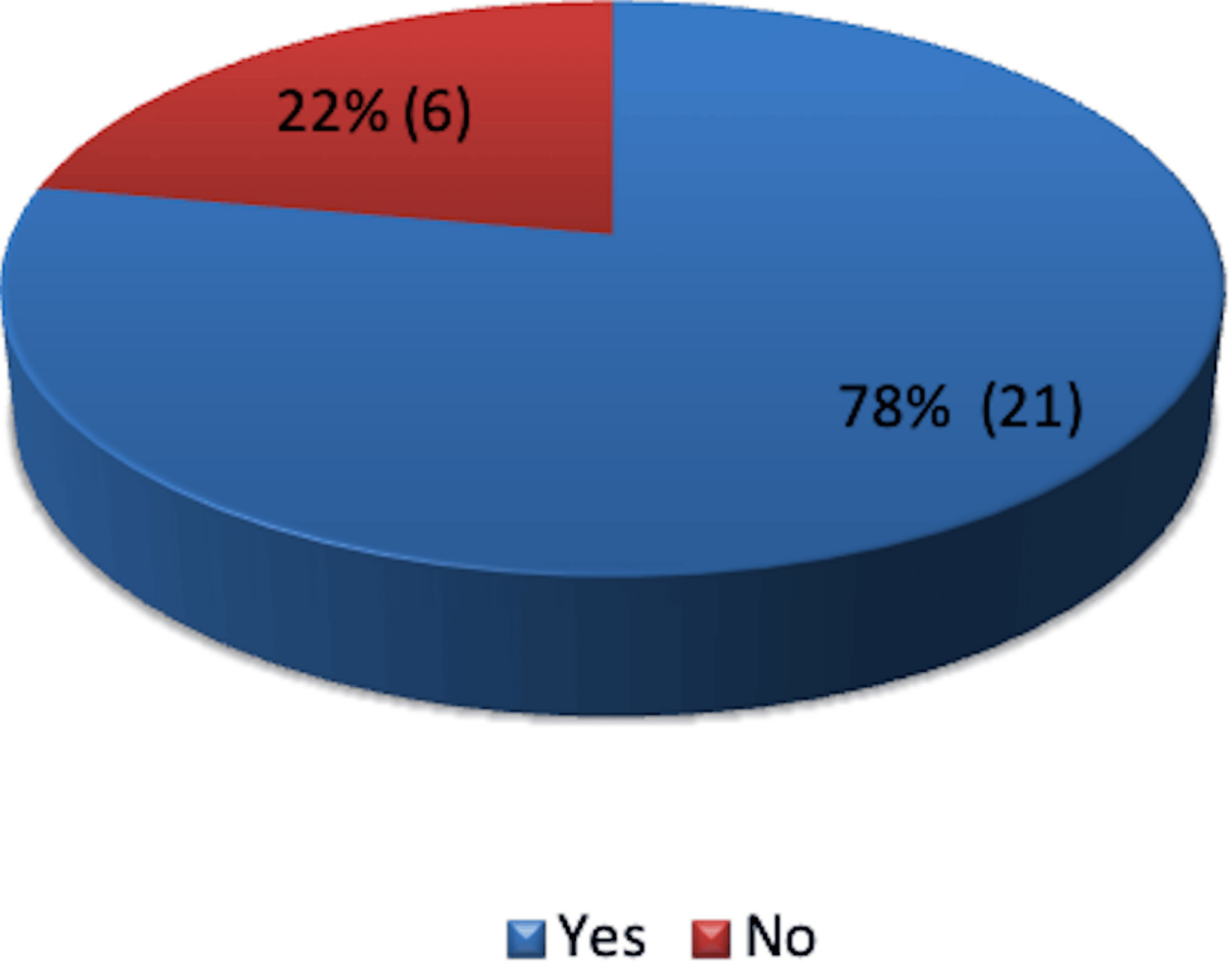
Emergency laparotomies - Yes or No

Of the 27 cases, 21 were emergency laparotomies, and sx were elective cases, including three cases, which were converted from laparoscopic procedures to open surgery (Figure 3).

**Figure 4:**
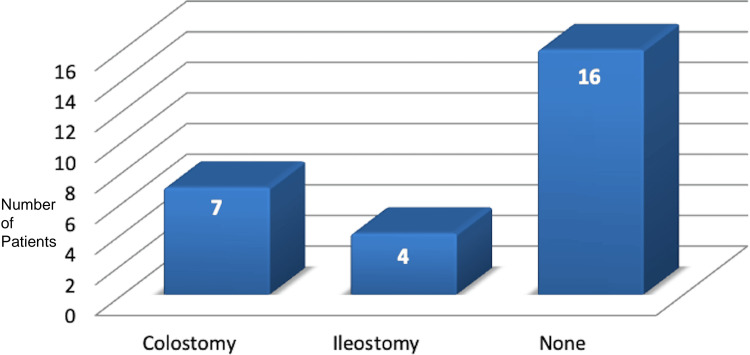
Types of stoma

Post-laparotomy, 11 patients required a stoma (seven colostomies and four ileostomies), while 16 patients did not require a stoma (Figure 4).

**Figure 5:**
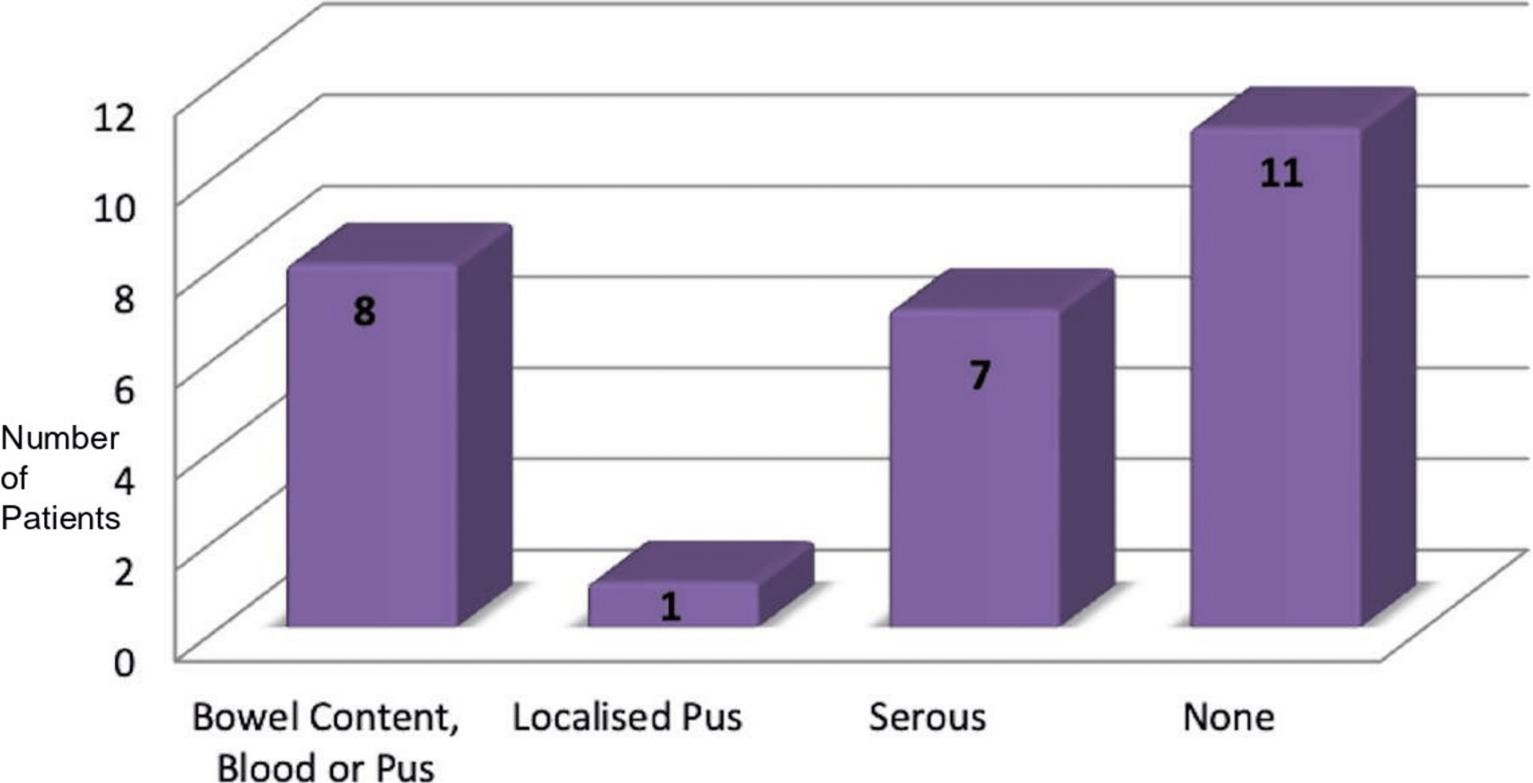
Type of peritoneal soiling

Peritoneal soiling was observed in eight patients, with contamination by blood, pus, or bowel content. Serous soiling was noted in seven patients, and 11 patients had no peritoneal contamination (Figure 5).

**Figure 6:**
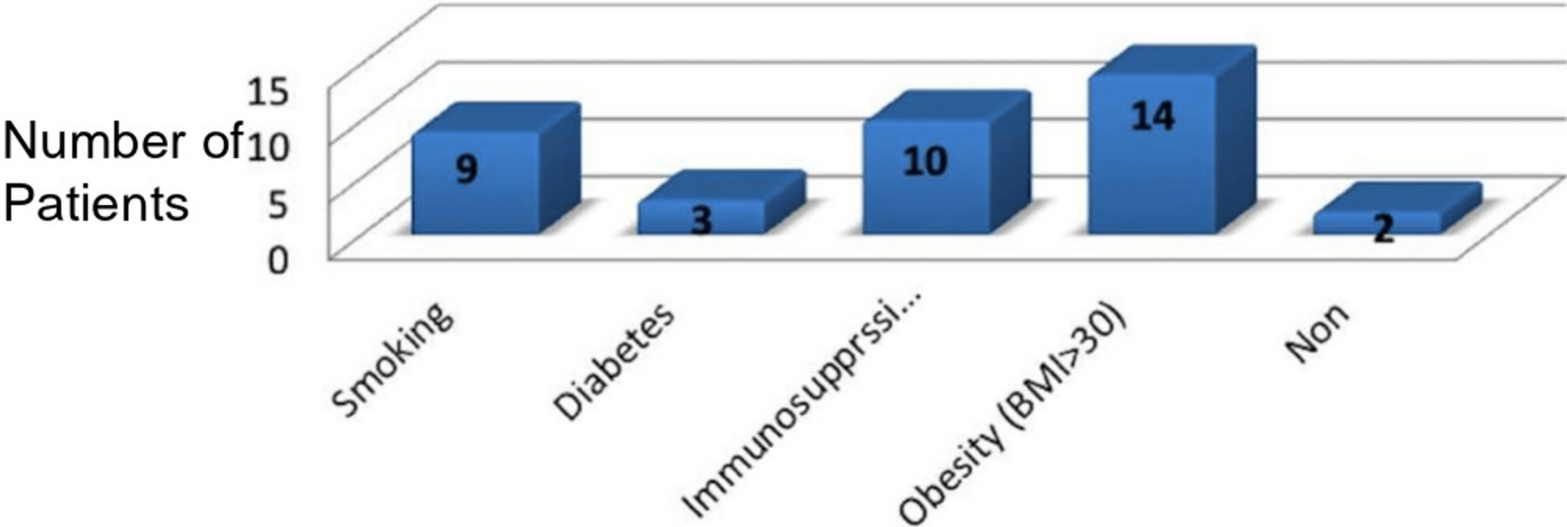
Risk factors for surgical site infection

The most significant risk factor was obesity, with 14 patients (over 50%) having a BMI above 30. Additional notable risk factors included immunosuppression (10 patients) and smoking (nine patients) (Figure 6).

**Figure 7:**
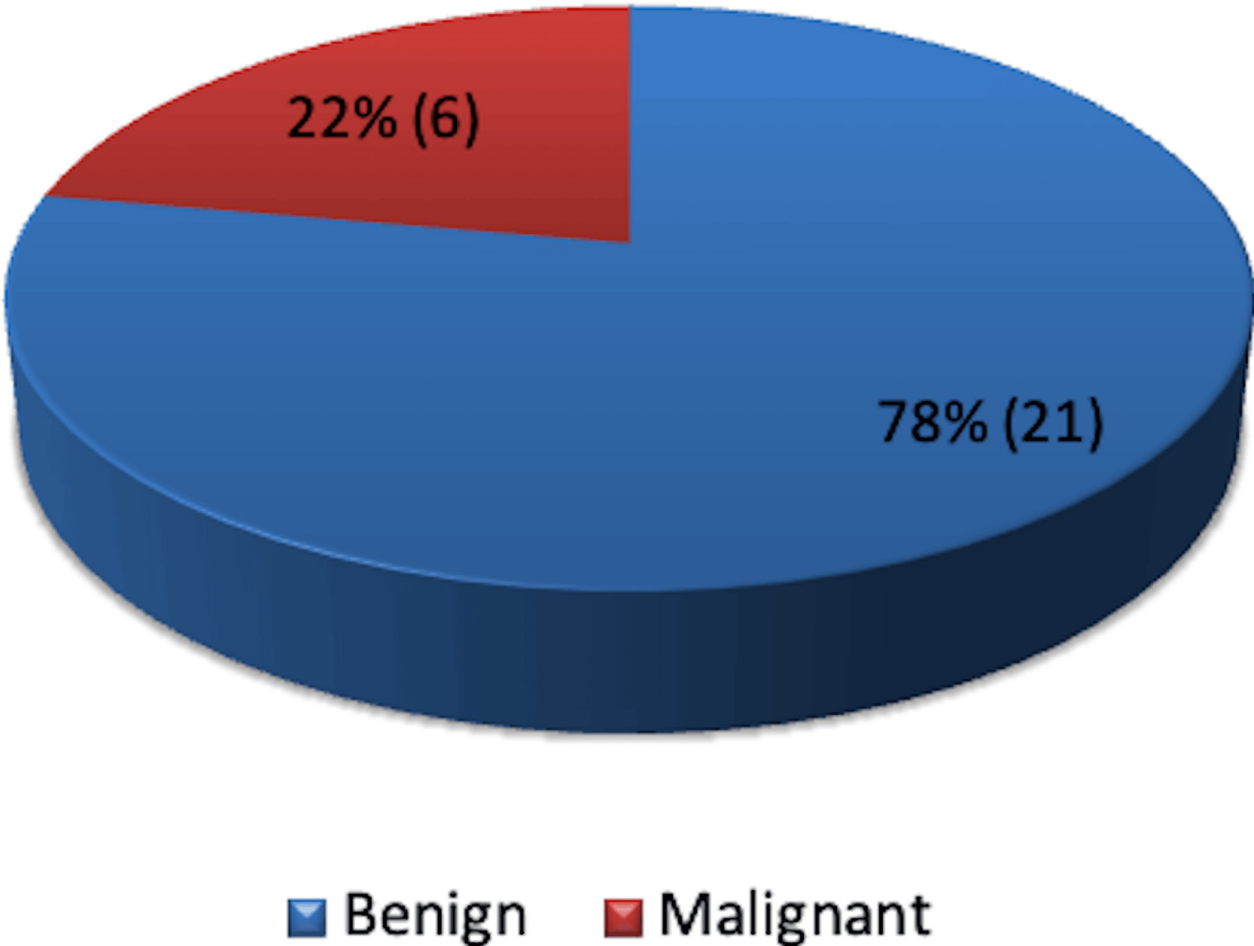
Pathology - benign or malignant

The majority of patients (21) had benign disease, while six patients had malignant pathology confirmed by histology (Figure 7).

**Figure 8:**
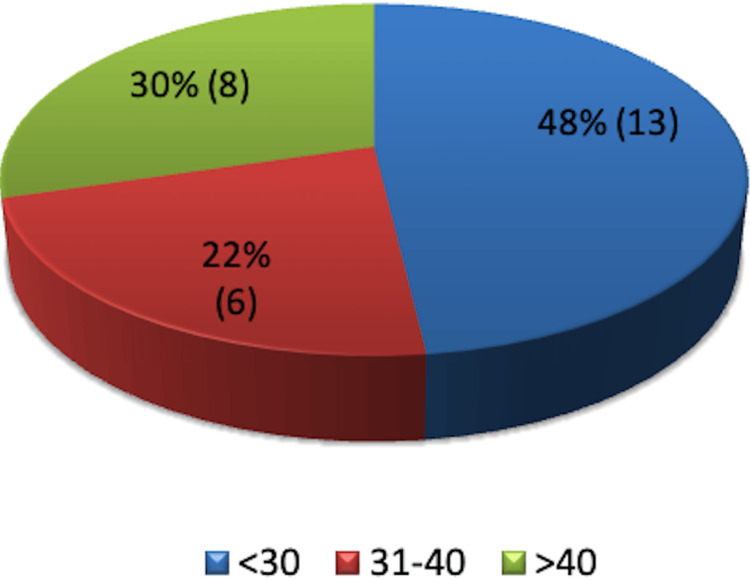
Body mass index distribution

More than 50% of patients were obese: eight had a BMI above 40, six had a BMI of 31-40, and 13 had a BMI under 30 (Figure 8).

**Figure 9:**
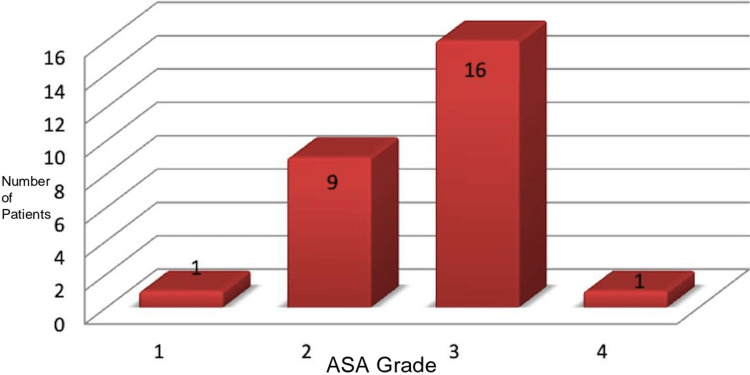
ASA grade distribution ASA - American Society of Anesthesiologists

According to the American Society of Anesthesiologists (ASA) physical status classification, 63% of patients were ASA grade ≥3. The majority (16 patients) were ASA grade 3, nine were ASA grade 2, and two were ASA grade 1 or 4 (Figure 9).

**Figure 10:**
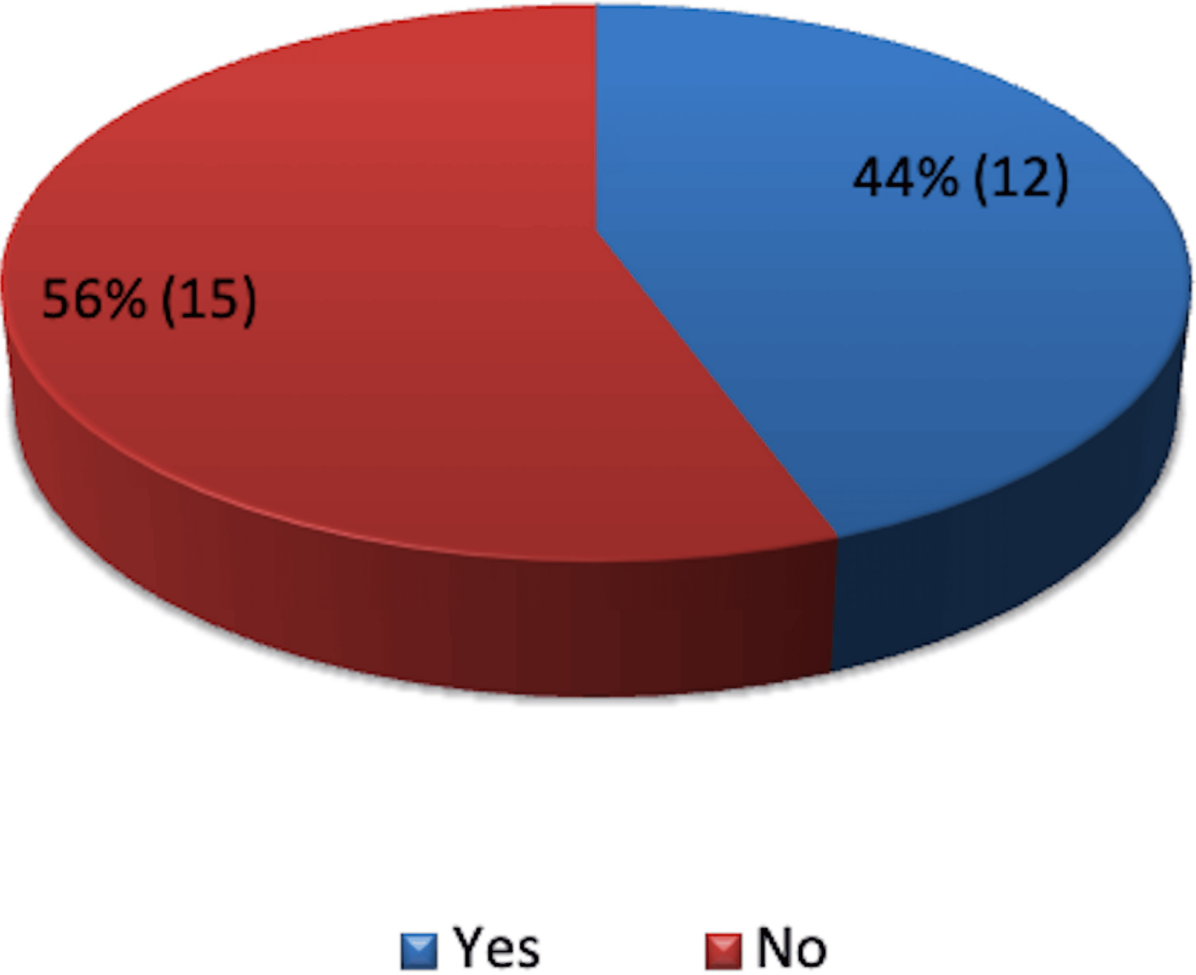
Removal of PICO dressing before seven days

All patients experienced blood loss of less than 100 mL during surgery. PICO dressings were removed before the recommended seven-day duration in 12 patients, while in the remaining 15 patients, they remained for the full period (Figure 10).

**Figure 11:**
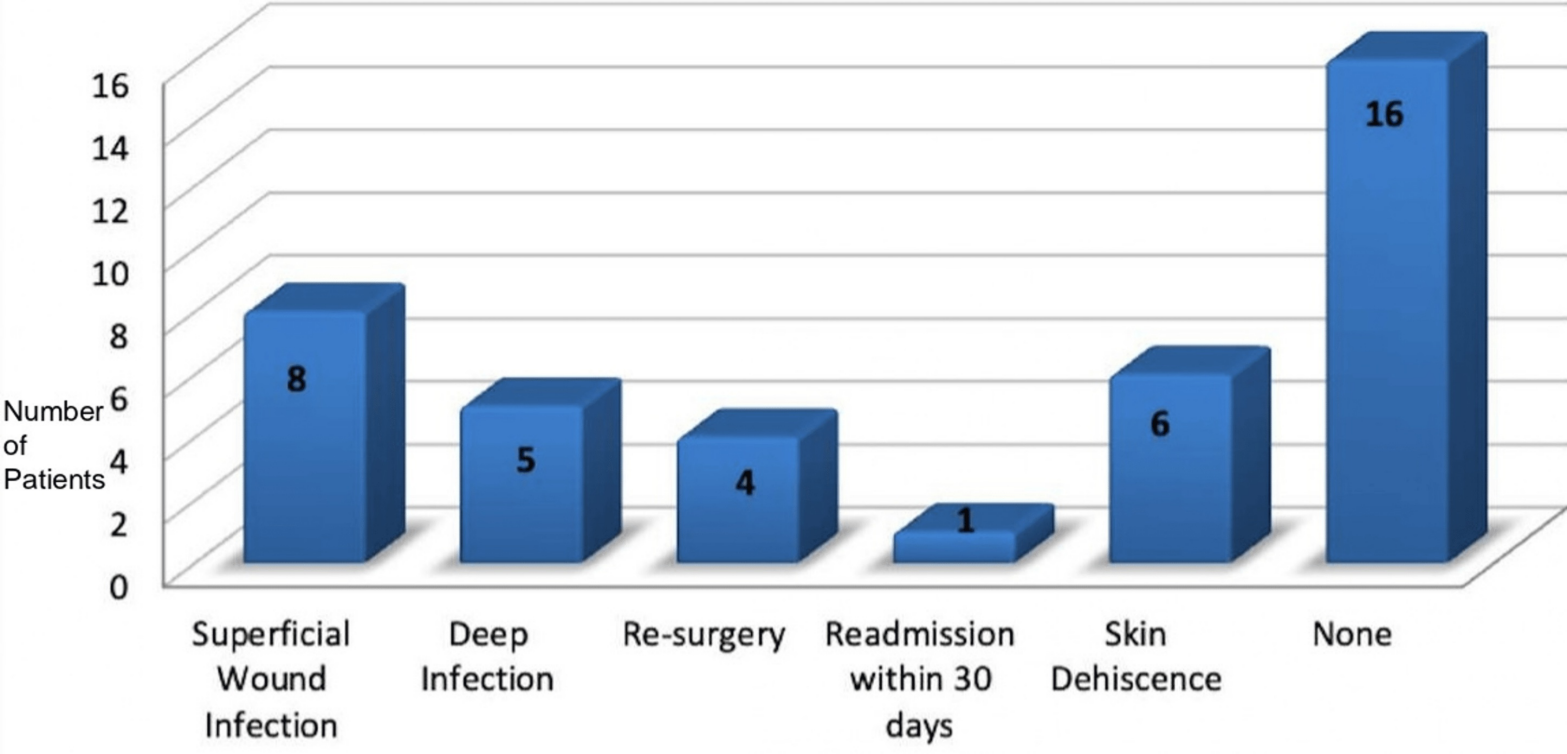
Spectrum of complications

Complications were absent in 16 patients (59.3%). The most common complication was SSI, observed in eight patients (29.6%). Skin dehiscence was noted in six patients (22.2%), and four patients (14.8%) experienced deep infections. One patient required a return to the operating room. Overall, 61.5% of patients did not develop SSI, while 38.5% did (Figure 11).

**Figure 12:**
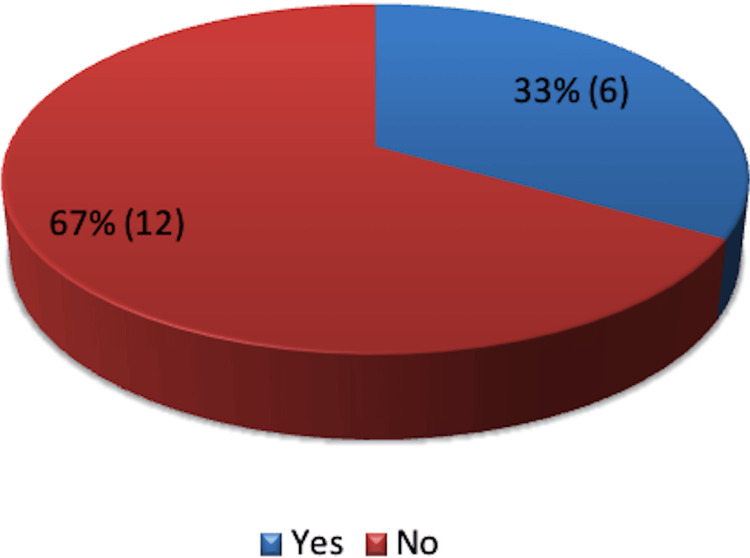
Positive wound cultures

Wound cultures were taken from 18 patients, with six testing positive for infection. Cultures were taken only if there was suspicion of SSI upon removal of the PICO dressing (Figure 12).

**Figure 13:**
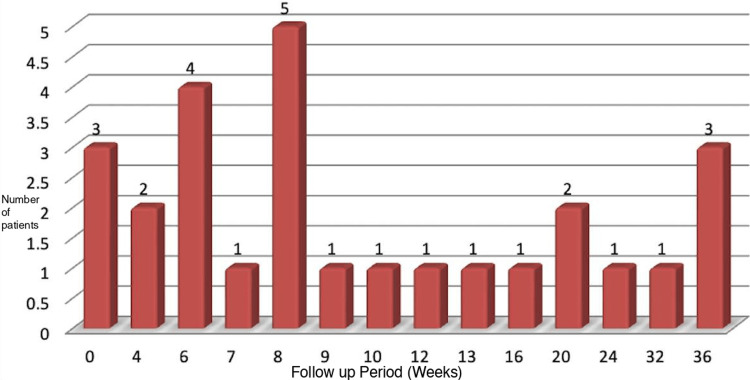
Post-operative follow-up period

The average hospital stay was 17 days, ranging from one to 74 days. The average follow-up period was 13 weeks, with the lower limit due to early mortality in one patient (Figure 13).

## Discussion

The focus of this study was to ascertain the impact of negative pressure wound therapy in the form of the PICO 7 dressing on SSIs. We analysed the data from our general district hospital and compared this with the literature. The study design was to have a comparison with a similar cohort of patients with normal conventional dressing at the same institute. However, due to the SARS-COV-2 pandemic, data collection was hampered. Therefore, it was decided that we would compare the institutional results with existing literature.

The primary endpoint of the study was to identify the rate of SSIs after placing negative pressure wound dressing, which would be identified in the form of purulent discharge from the wound: culture-positive wound swab, localised signs of acute inflammation such as rubor (redness), dolor (pain) and calor (heat). Of the 27 cases, one had to return to theatre twice and had PICO 7 applied twice. Of the actual 26 patients, 16 (61.5%) patients had no surgical wound complications. Amongst the remaining 10 patients (38.5%), eight (30%) had SSI with overlap in the form of dehiscence and deep space infection, and two patients had isolated deep space infection. Looking at individual wound complications among these 10 patients, eight had superficial SSI, five had deep space infection, six patients developed skin dehiscence, four had further surgery/percutaneous drainage for collection, and one patient had readmission in 30 days with a single patient having more than one complication.

Strugala et al. carried out a meta-analysis which included 16 studies with 1,863 patients and studied the impact of negative pressure wound therapy (PICO) on surgical site complications [13]. They included studies with orthopaedic, abdominal and caesarean operations. Their meta-analysis demonstrated a significant reduction of risk of SSI from 12.5% with standard dressing to 5.2% with PICO and a reduction in wound dehiscence from 17.4% with standard dressing to 12.8% with PICO 7. Amongst these 16 studies, 10 were randomised controlled trials, and six were observational studies. However, only three studies dealt with colorectal surgery. In comparison to the above meta-analysis, the risk of SSI in this study is 38.5%, which is significantly higher than existing literature reports.

There are several factors contributing to a high rate of SSI with PICO 7 in this study, as compared to that reported in the existing literature. Firstly, the meta-analysis by Strugala et al. included all types of surgeries, such as orthopaedic surgery, caesarean section, and abdominal surgeries [14]. Caesarean sections and orthopaedic surgeries are not at high risks for wound infection as compared to emergency abdominal surgeries, which are contaminated with faecal matter and pus. Therefore, the majority of data in this meta-analysis came from the outcomes of clean surgeries, reflecting an overall lower rate of SSIs, not only with PICO dressings, but also with standard dressings. Consequently, the rate of SSI would be high in studies, which include exclusive emergency abdominal surgeries (e.g., this current study), as compared to studies, which include non-abdominal surgeries.

Secondly, intra-abdominal surgeries involve bowel resection and anastomosis. Therefore, in addition to the usual risk factors for SSI, such as high BMI, immunosuppression, and diabetes mellitus, abdominal surgeries include the significant additional risk of SSI secondary to anastomotic leaks. An anastomotic leak can present in the form of deep space infection, superficial SSI, and wound dehiscence. This could result in a return to theatre for further surgery, wound wash out and debridement, re-suturing of dehiscence, and percutaneous drainage of deep space infection.

Winfield et al. carried out an analysis of over 89,000 patients who underwent an abdominal procedure [15]. They found that obese and morbidly obese patients were at a significantly greater risk of developing SSI, specifically in clean and clean-contaminated operations. Obesity is generally associated with other co-morbidities such as type 2 diabetes mellitus, as well as atherosclerotic vascular disease, which contribute to impaired wound healing. Additionally, the thickness of subcutaneous fat is indicative of the risk of SSI following a surgical procedure [16]. This was further evidenced in a meta-analysis by Gurunathan et al. who specifically identified that overweight and obese patients had 20-50% higher odds of contracting an SSI following an abdominal surgery [6]. Obesity not only reduces the responsiveness of lymphocytes and alters the function of the immune system but also affects the tissue oxygen demand and supply, thus leading to reduced oxygen tension in the wound and increasing the chances of wound dehiscence. Our study found that 50% of our surgical candidates were overweight or obese, and a particular patient, with a BMI of over 40, suffered from SSI, skin dehiscence, and subsequent return to theatre for debridement. This is in keeping with the literature.

Another significant factor, which increases the risk of SSI in emergency laparotomies, is that emergency abdominal surgeries often result in the formation of a stoma. Stoma formation leads to faecal matter passing through the abdominal wall, which in itself is enough to contaminate the abdominal wall wound. This can occur when faecal contents accidentally seep into the main laparotomy wound while changing the stoma bag. A single accident of spillage of faecal contents while changing the stoma bag can result in contamination of the abdominal wound, greatly increasing the risk of SSI. This brings the human factors into play. Therefore, specialist support from colorectal stoma care nurses, appropriate training of patients and their relatives for changing the stoma bag, and their competence to do the task independently without spillage of contents are of paramount importance in avoiding SSIs.

Furthermore, if the vacuum seal fails or leaks, non-sterile products can contaminate the wound bed and create a fertile ground for infections. The proximity of the stoma to the wound can act as a source of contamination for the negative pressure wound environments too and may amplify the effect of a vacuum leak. In the current study, 16 patients had some form of abdominal cavity contamination in the form of faecal matter, pus, or serous collection. As soon as the abdominal cavity is opened, the abdominal wall comes in contact with these contaminants, and the risk of SSI is greatly increased, as compared to other surgeries where such contamination is absent, inducing the bias for high infection rates. Additionally, 11 out of 27 laparotomies in this study were accompanied by stoma formation, with seven of them having colostomy and four had ileostomy. As discussed earlier, the stoma poses a significant challenge in terms of the risk of SSI, as bowel contents overflow into the laparotomy wound and significantly increase the risk of SSI. Although PICO 7 forms an air tight negative suction and should not allow seepage of faecal contents into main wound, faecal flora can still potentially migrate close to PICO 7, and its negative suction effect can suck these micro-organisms into the main wound. This can result in devastating SSI. A study by Webb et al. supports the argument that stoma formation increases the risk of SSI with the use of NPWT. They observed an independent association of NPWT with SSI and showed 17% SSI with NPWT in comparison to 9% SSI with standard conventional dressings, in patients who underwent stoma formation in emergency abdominal surgery.

The other factor contributing to the high SSI rates in this study was the effect of immunosuppression. Immunosuppression has been repeatedly shown as an independent risk factor for developing SSIs regardless of the procedure. Conditions such as diabetes and malignant pathology are known to induce immunosuppression. In this study, approximately a quarter of patients had malignancy as a predominant pathology, affecting six patients, and the rest of 21 patients had benign pathology. Malignancy is an immunosuppressive state, and these patients are at significant risk of wound breakdown, anastomotic leak, and SSI because of impaired immunity. Furthermore, a small cohort of our patients also suffered from diabetes, which creates an immunosuppressive state and may contribute to the higher risk of SSI, regardless of the use of NWPT. Poor post-operative glycaemic control (48-h MCG >11.0 mmol/L or 200 mg/dL) has been reported to be an independent risk factor for increased SSI following colorectal resection in diabetic patients [17].

Moreover, after carefully analysing the results, it was noted that the predominant setting for the application of PICO 7 in this study was emergency. In 78% (21 out of 27) of the cases in this study, PICO 7 was applied in the emergency setting, whereas only 20% (six out of 27) cases had PICO applied in elective setting. Emergency surgery is always considered a high risk for SSI because of the inherent nature of the urgency of surgery and associated physiological derangements. Generally, there is suboptimal optimisation of patient physiology in the form of inadequate glycaemic control, lack of normothermia because of long procedure duration, and insensible losses of fluids from body surfaces. Not only the abdominal milieu may be found contaminated, but emergency surgery does not have any bowel preparation, which may contribute to a higher incidence of SSI, despite the use of PICO 7 dressing. Additionally, in emergency settings, damage control is the main objective, where the surgeon aims to quickly finish the procedure and shorten the anaesthetic duration, in order to prevent complications because of fragile physiology. Therefore, in such states, sometimes effective abdominal wall closure is less important than getting the patient off the operating table for the physiological status to stabilise. As a result, emergency patients tend to develop more short- and long-term wound complications, such as SSI and incisional hernia, respectively. Since this study has a majority of emergency patients, they might tend to be affected by the nature of the surgery, where life-saving quick procedures take precedence over slow meticulous abdominal wall closure.

SSIs are one of the most common complications in the post-operative patient. An SSI is defined as an infection of the wound that occurs within 30 days of the operation. The infection subsequently translates into increased morbidity, mortality, and prolonged hospital stay, adding to higher costs and poorer outcomes. Alkaaki et al. studied 337 patients for SSIs and found an overall incidence of 16.3% [1]. The independent factors that contributed to the risk of acquiring an SSI included open surgeries, emergency operations, and prolonged time in the operating theatre. In their prospective study, they found that SSI was 4.8 times more likely to occur in emergency settings as compared to elective procedures. The length of the operation was also of statistical significance, with an odds ratio of 2.1. The laparotomies that were performed on the patients at our DGH were nearly always in an emergency setting. The details on the length of the operation were, however, not collected in our study but are a consideration for future studies.

Secondary endpoints of the study were the length of stay, readmissions, and return to theatre with intervention in the form of radiological or open drainage of any collection. In this study, the average length of stay was 13 days, one patient had readmission with SSI, and four patients had interventions in the form of return to theatre or radiological drainage for deep space collection. Curran et al. observed the mean length of stay was 8.7 days in patients with NWPT, and 8% of patients had unplanned readmission [2]. There are several reasons for the longer length of stay in the current study. The majority of the patients (78%) in the study underwent emergency surgery, with nearly 60% of patients having contaminated abdominal cavities with faecal contents, pus, blood, or serous fluid. In comparison, the cases studied by Curran et al. consisted of elective surgeries in 70% of cases. In cases with elective surgery and uncontaminated peritoneal cavity, the rate of SSI will undoubtedly be lower, hence explaining the shorter length of stay in their study.

In our study, several factors contributed to the longer length of stay. Firstly, the majority of cases were emergency surgeries, which involved major bowel resection in a contaminated milieu. As a result, a lot of patients developed post-operative complications, such as anastomotic leak and hospital-acquired pneumonia. In the background of immobility, malignancy, and obesity, pulmonary embolisms also contributed to the prolonged hospital stay. Secondly, a heavily contaminated abdomen resulted in post-operative wound complications and sepsis, which led to a longer duration of stay. Thirdly, because of the urgency of surgery in an emergency setting, there was very little time to optimise the patients before proceeding to laparotomy. Consequently, patients with a poor physiological reserve the required post-operative optimisation and care in the critical care unit. Eventually, this translated into patients staying over for a longer duration of time than expected.

Approximately 63% of the patients were ASA 3 and above, which translates into severe systemic disease. When taking into consideration the National Emergency Laparotomy Audit (NELA) score, it was evident that the prognosis was not optimistic. Moreover, >50% of patients were obese, which further affects the recovery and increases the potential length of stay. A significant proportion of patients had malignant pathology and were immunosuppressed. Complex intrabdominal surgery with malignancy in an emergency setting is a high-risk surgery and patients need optimum support before they can go home. Approximately 40% of patients had stoma formation in an emergency setting. Stoma training and support by specialist colorectal nurses is extremely important for the patients and their families before they can go home and manage the stoma on their own confidently. Consequently, training in stoma and its management itself added a few days to the length of stay.

Finally, social factors played a crucial role in expediting the discharge of patients to home. Within Britain’s National Health Service, social services and therapy services play a crucial role in supporting patients in the community setting. Patients with good family support and modest functional status can be sent home on their own. However, the elderly age group who live alone or within care homes need appropriate support before returning to community care. As a result, a lot of patients had to stay in hospital, despite being medically optimised for prolonged periods of time. In some cases, these patients would develop hospital-acquired infections, including pneumonia, urinary tract infections, and Clostridium difficile infections. With the extreme burden on elderly social care and rehabilitation services, patients tend to overstay despite being medically optimised. Unfortunately, in a few circumstances, patients developed urinary tract infections and hospital-acquired pneumonia while awaiting care package, which was treated subsequently. This resulted in further prolongation of the duration of stay. Thus, our length of stay does not directly reflect the effect of the rate of SSI or consequences of SSI, but a combination of complex physiological, pathological, and social factors.

The study is limited by several shortcomings. It is mainly an observational study and is therefore prone to selection and reporting bias. There was no use of an externally validated calculator such as the NSQIP surgical risk calculator. The use of an externally validated risk calculator could have effectively predicted actual SSI rates. Outcomes of NPWT were determined on the basis of case notes, and it was dependent on the clinical judgement of the surgeon and the nurses. Moreover, a lot depends on proper documentation on the part of the attending clinician and their team. Therefore, owing to several variables, it is easy to have reporting bias which might affect the outcome of the study.

Although initially it was proposed in the study design that a control group would be used to look for SSI with the use of the standard surgical dressing, however, this has not been done. Due to the COVID-19 pandemic, all elective and academic activities were disrupted. Moreover, to observe social distancing and government guidelines of staying at home, the audit department and team were working off-site. As a result, it was difficult to retrieve the operative notes, and with all the clinical work being diverted towards the COVID-19 pandemic, the research team could not complete the said task in the given time frame.

Additionally, the study group had a very small sample size owing to the constraints of time and resources. A small sample size increases the chances of type I error, and the results will be definitely affected by selection bias. To have an appropriate study with statistically significant results, an adequate sample size needs to be calculated. The sample size should be large enough to reveal a statistically significant effect of the intervention, whose results can be considered relevant for the population in question. This further needs to be compared to a matched control cohort with the equal number of participants, for good-quality results. Unfortunately, this could not be done due to the aforementioned limitations.

While analysing the data, we realised that deep space infection should not have been included in the rates of SSI in the study design. Deep space infections are influenced by the occurrence of anastomotic leak, the degree of pre-operative peritoneal soiling, and the adequacy of peritoneal lavage. Meanwhile, NPWT only affects the incidence of superficial SSI related to the wound and will make no difference to the prevention of deep organ space SSI. In fact, in patients with standard surgical dressing or PICO 7 dressing, the rates of SSI would be similar. Hence, while quoting the rate of SSI, our study erroneously overestimated the rate of SSI, rather than reflecting what should have been the exact value.

Another shortcoming of the study design that became evident while evaluating results is that the length of stay does not reflect the impact of SSI. Several factors were responsible for prolonged length of stay, which were not taken into account while collecting the data. Most of the cases were emergency surgical procedures, which needed post-operative optimisation of physiology and support of critical care. In the post-operative period, patients needed support from colorectal nurses regarding stoma care. Additionally, the majority of cases were elderly patients, who needed support from social services and waited for care plans and therapy services to sort out the home situation before they could be stepped down. Therefore, the length of stay was dependent on multiple factors, such as the nature of surgery, pathology, postoperative care, and social support. They had a confounding effect on the rates of SSI. Therefore, attributing our length of stay completely to SSI does not reflect the true causation.

Furthermore, in the study, the sample size was quite small to have a significant effect in terms of validity. However, within the time constraints and financial feasibility of the study, it was the best that could have been done. However, a bigger study group with a control group of standard surgical dressing will give more appropriate and statistically significant results, and such a study can be contemplated in the future.

## Conclusions

SSIs remain a significant postoperative complication following abdominal surgeries. The use of NPWT, such as PICO 7, has demonstrated the potential to reduce the risk of SSIs. Despite its application, approximately 30% of patients in our study developed SSIs, which can be attributed to factors such as perforated viscus, high BMI, multiple comorbidities, and stoma creation near the wound site. Comparative data from the literature, primarily involving elective procedures such as hip replacements and cesarean sections, show lower SSI rates of approximately 16%. A larger cohort study with a control group using conventional dressings and matching demographics is necessary to further evaluate the effectiveness of NPWT in abdominal surgery. The lack of a control group in our study, due to the SARS-CoV-2 pandemic, underscores the need for future research comparing NPWT and conventional dressings in similar surgical populations to better understand their impact on SSI rates.
